# Reliability and validity of the National Institutes of Health Chronic Prostatitis Symptom Index questionnaire in the Turkish Population

**DOI:** 10.3906/sag-2001-231

**Published:** 2021-04-30

**Authors:** Alper COŞKUN, Utku CAN, Fatih TARHAN, Övünç KAVUKOĞLU, Kamil Fehmi NARTER

**Affiliations:** 1 Department of Urology, University of Health Sciences, Kartal Dr. Lütfi Kırdar Training and Research Hospital, İstanbul Turkey; 2 Department of Urology, Acıbadem Mehmet Ali Aydınlar University Kadıköy Hospital, İstanbul Turkey

**Keywords:** National Institutes of Health Chronic Prostatitis Symptom Index (NIH-CPSI), chronicprostatitis/chronicpelvicpainsyndrome (CP/ CPPS), Turkish validation

## Abstract

**Background/aim:**

To develop the first Turkish version of the National Institutes of Health Chronic Prostatitis Symptom Index (NIH-CPSI) questionnaire and to investigate its validity and reliability in men with chronic prostatitis/chronic pelvic pain syndrome (CP/CPPS) and healthy controls.

**Material and methods:**

A total of 204 patients, 116 CP/CPPS and a control group which consisted of 88 healthy individuals were included in this prospective study. The NIH-CPSI form was first translated into Turkish and later on back into English. Using the Turkish version of the NIH CPSI, 40 patients completed the same questionnaires twice at 2-week intervals for test-retest concordance. To evaluate internal consistency and test-retest reliability, Cronbach’s alpha value, and the Spearman correlation test were utilized respectively.

**Results:**

Our findings demonstrated statistically significant differences in NIH-CPSI scores between the patients and control groups (P <0.001). Cronbach’s alpha coefficient value of NIH-CPSI was 0.864. Reliability of test-retest was 0.909 (P <0.001). Additionally, the Spearman correlation test showed that the results obtained using the Turkish NIH-CPSI were significantly correlated.

**Conclusion:**

The first Turkish version of the NIH-CPSI was found to be a reliable and valid instrument for Turkish patients with chronic prostatitis in both clinical and research settings.

## 1. Introduction

Despite the higher prevalence of benign prostate ichyperplasia (BPH) and prostate cancer in men older than 50 years; prostatitis is the most frequently diagnosed urological problem in men younger than 50 years and the third most common urologic diagnosis again in cases with an age value of >50 years [1].**** Chronic prostatitis/chronic pelvic pain syndrome (CP/CPPS) is a common urological disease characterized by the limited response to conventional management approaches and has a complicated healing process. [2] Men suffering from chronic CP/CPPS are the largest population being evaluated in outpatient clinics. The incidence of the pathology ranges from 5% to 14.2% [3,4] and relevant research studies have shown that approximately 16% of men may present with a prostatitis attack at least once in their lifetime [5].

The National Institutes of Health (NIH) classification describes four patterns of prostatitis; type 1 acute bacterial prostatitis, type 2 chronic bacterial prostatitis, type 3 CP/CPPS, and type 4 asymptomatic prostatitis [6]. CP/CPPS has been defined with pelvic pain noted in at least three of the previous 6 months with no other underlying causes detected. Additionally, the NIH subclassifies type 3 as inflammatory chronic pelvic pain syndrome (3a) and noninflammatory chronic pelvic pain syndrome (IIIb) [5].

CP/CPPS is associated with certain symptoms including pain (during urination and in the abdomen, lower back, rectum, and genital area), dysuria, urinary urgency, perineal discomfort, obstructive or irritative voiding difficulties, and hematospermia. Pain is the predominant characteristic symptom of CP/CPPS and it is not only associated with disturbed quality of life but also significant sexual dysfunction [7–9]. Diagnosis of the pathology is usually made based on the symptoms stated where the duration and persistence of these symptoms constitute the first step in the evaluation of CP/CPPS. The National Institutes of Health Chronic Prostatitis Symptom Index (NIH-CPSI) is a commonly used tool to diagnose CP/CPPS, to evaluate the severity of symptoms, and to provide available rational treatment options.

**Figure 1:**
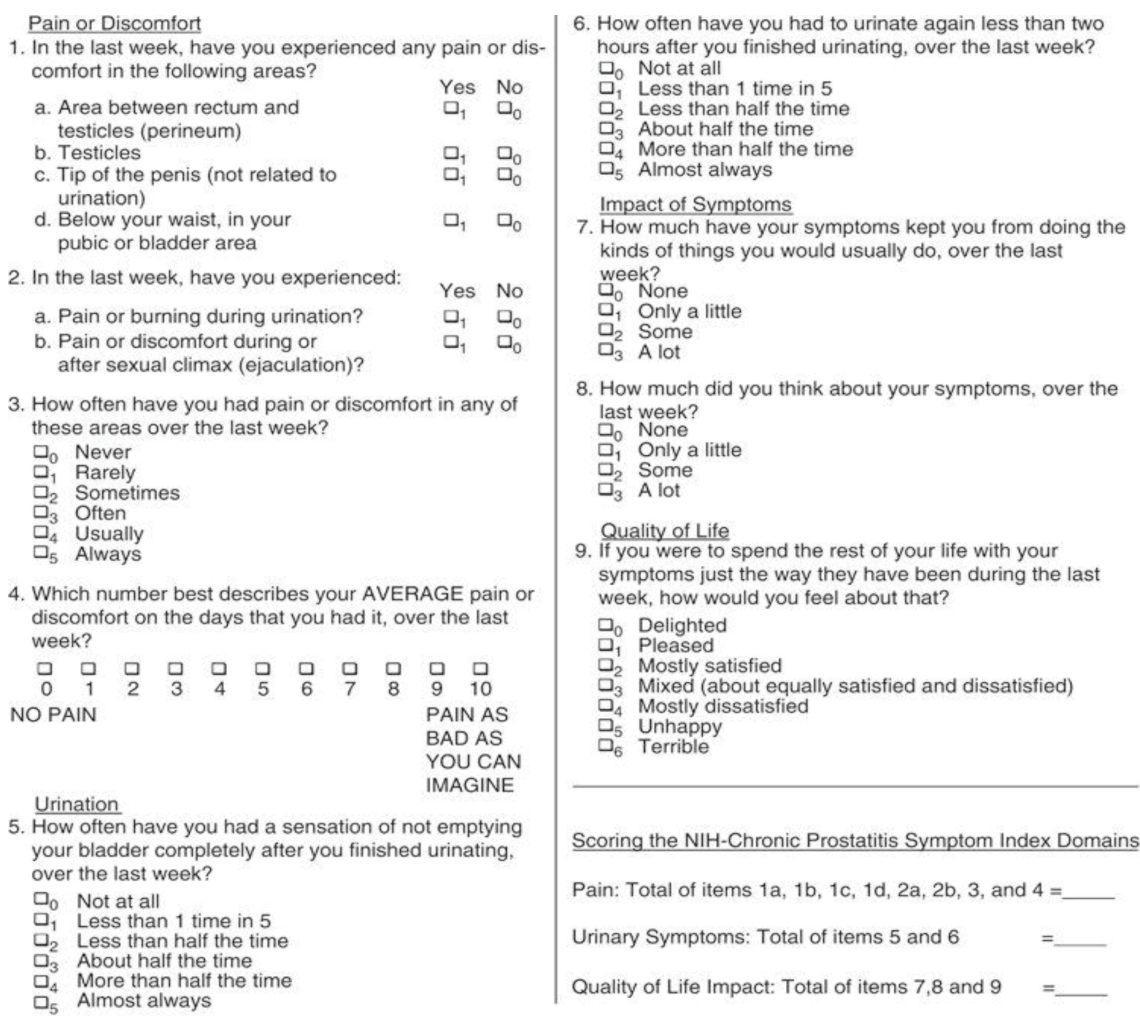
NIH Chronic Prostatitis Symptom Index (NIH-CPSI).

The form consists of three subscales with a total score ranging from 0 to 43: pain or discomfort (with a total score ranging from 0 to 21), urinary symptoms (with a total score ranging from 0 to 10), and impact on the quality of life (QOL) (with a total score ranging from 0 to 12 points [1]. Pain scores of perineal or ejaculatory discomfort ≥8 are good predictors of patients with moderate to severe CP/CPPS [10]. It has been shown that as the symptoms get more severe, the scores will also be higher (Figure 1).

The translation and linguistic validation of the NIH-CPSI into various languages was made previously in a successful manner [1,4,11,12]. In the present study, we mainly aimed to form and validate the Turkish version of the NIH-CPSI. In addition, we also investigated whether the patients’ responses to urinary symptoms were compatible with the International Prostate Symptom Score (I-PSS) and Visual Analogue Scale (VAS) evaluations [13,14].

## 2. Materials and methods

### 2.1. Study design

The study included 116 CP/CPPS patients referring to Dr. Lütfi Kırdar Kartal Training and Research Hospital Urology Department with recurrent symptoms and no response to conventional management approaches for at least 6 months and 88 healthy controls. Patients with psychiatric disorders, cancer history, benign prostatic hyperplasia, and other severe comorbidities were all excluded from the study program. All of the participants completed the Turkish NIH-CPSI by themselves. Additionally, they were also asked to complete the IPSS and VAS regarding their urinary symptoms. To evaluate the test-retest reliability, 40 patients were asked to recomplete the NIH-CPSI once again two weeks later. The study protocol was approved by the ethics committee of our hospital, and all the participants were asked to sign a detailed informed consent stating all procedures in line with the Helsinki Declaration (193/2013).

### 2.2. Translation process

**Figure 2:**
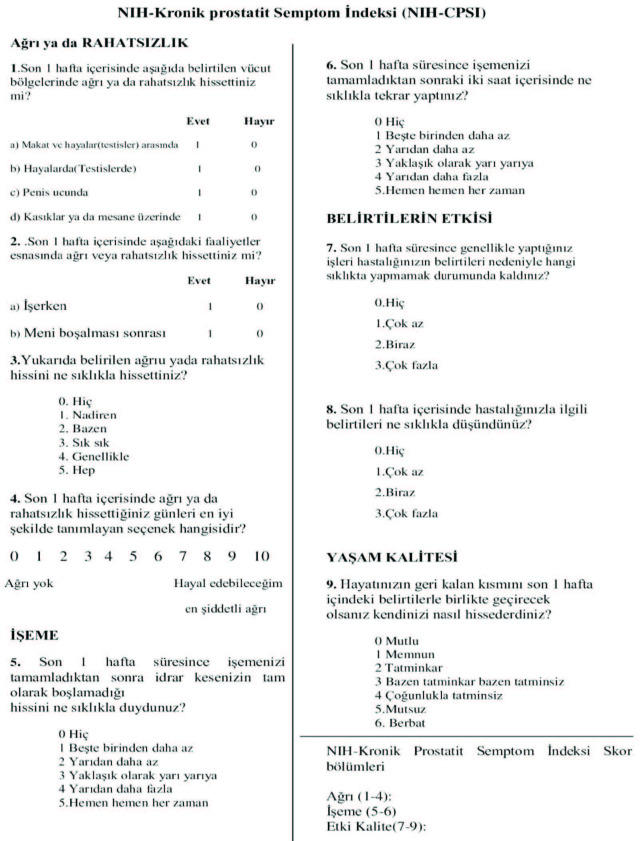
Turkish NIH Chronic Prostatitis Symptom Index (NIH-CPSI).

To perform the translation process, the multistep validation method defined by Hutchinson et al. was used as a guide [15]. These steps are outlined below:

1. The questionnaire was translated from English to Turkish by two independent translators with proper skill and fluency in speaking both English and Turkish.

2. To compare and evaluate the translated Turkish forms, a meeting was held between the research group and the two translators.

3. The final form of the questionnaire on which a consensus has been reached was translated back into Turkish language by the two translators simultaneously, regardless of the source of the original questionnaire.

4. Minor inconsistencies were resolved, and the final form was given during the second meeting between the translators and the researchers.

5. Finally, in an attempt to determine whether the final version of the questionnaire was appropriate and easily understandable, a pilot study was carried out with the inclusion of 15 symptomatic patients demonstrating the highest NIH-CPSI symptom scores. This study demonstrated the final questionnaire to be understandable and the final updated form was approved without any changes (Figure 2).

### 2.3. Statistical analysis

The general characteristics of the participants were evaluated using descriptive statistics, and the data were presented as mean ± standard error of mean (SEM). To assess the psychometric analysis of NIH-CPSI, reliability, internal consistency, and test-retest reliability were taken into consideration. Internal consistency was evaluated using the Cronbach alpha value, while test-retest reliability was evaluated using the Spearman correlation test. Discriminant validity was evaluated based on the information obtained by comparing the results of the patients with those of the control group. The Mann–Whitney U test was used to investigate potential differences and similarities between the groups. All statistical analyses were performed using SPSS 22.0 (IBM® SPSS® Statistics V22.0, 2013, USA), and P <0.05 was considered statistically significant.

## 3. Results

A total of 116 CP/CPPS patients and 88 healthy controls were included in the study program and the demographic characteristics of the patients are shown in Table 1. The mean age of the CP/CPPS and control groups were 38 and 39.4 years, respectively (P = 0.519). The mean duration of the symptomatic status in CP/CPPS group was 12 months. There was a significant difference regarding the NIH-CPSI (both the overall scale and subscales) scores of both groups (P < 0.001) (Table 1). The Spearman correlation test supported the significant correlation of our Turkish NIH-CPSI results. Cross-tabulation with correlations between the VAS, I-PSS, QOL, total NIH-CPSI and its subscales are demonstrated in Table 2.

**Table 1:** Baseline characteristics: age, duration of symptoms, NIH-CPSI scores, VAS, I-PSS, and QOL in the study groups.

	CP/CPPS	Controls	P*
No. of patients	116	88	
Mean age (SEM)	38 (9.3)	39.4 (10.7)	0.519
Duration of symptoms, month (range)	12 (16–240)		
Mean score of NIH-CPSI (SEM) Total (0-43)	22.8 (7.7)	2.5 (1.7)	<0.001
Pain (0–21)	9.5 (4.8)	1.4 (1.2)	<0.001
Urinary symptoms (0–10)	6 (3.2)	0.6 (0.8)	<0.001
Life quality impact (0–12)	7.3 (2.3)	0.5 (0.6)	<0.001
VAS (0–10)	4.5 (1.8)	1.2(0.3)	<0.001
I–PSS (0–35)	14.3 (8.5)	7.5(2.6)	<0.001

*The MannpWhitney U-test NIH-CPSI: National Institute of Health Chronic Prostatitis Symptom Index, VAS: Visual Analog Scale, IPSS: International ProstateSymptomScore, SEM: Standart eror of mean.

**Table 2:** Correlation matrix (Spearman) of VAS, I-PSS, QOL, and NIH-CPSI and its subscales among 116 patients with CPPS.

	NIH-CPSI Total	NIH-CPSIPain	NIH-CPSI Void	NIH-CPSIQol	VAS	IPSS
NIH-CPSI Total						
NIH-CPSI Pain	r = 0.77**					
NIH-CPSI Void	r = 0.72**	r = 0.33**				
NIH-CPSI Qol	r = 0.66**	r = 0.25*	r = 0.42**			
VAS	r = 0.6**	r = 0.66**	r = 0.22*	r = 0.29**		
IPSS	V0.48**	r = 0.24*	r = 0.54**	r = 0.4**	r = 0.25*	

**Correlation is significant at the 0.01 level.*Correlation is significant at the 0.05 level VAS: Visual Analog Scale,

The NIH–CPSI pain scores and VAS in the subgroup patients were found to be highly correlated (0.66). Moreover, the correlation between voiding scores and IPPS was found to be highly significant by reaching the value of 0.01 (r = 0.54) (Table 2). In test-retest evaluation, the total NIH-CPSI score (0.909) was found to be correlated well with the scores of the subgroups (P < 0.001). Lastly, the psychometric analysis has shown a great internal consistency with high Cronbach’s alfa coefficient values for the overall NIH-CPSI (0.864) (Table 3).

**Table 3:** Reliability of the test-retest analysis (Spearman) and internal consistency (Cronbach’s alpha coefficient) of the present study.

Domain	Cronbach’ salpha	Test	Retest	Correlation
NIH-CPSI total	0.864	22.8 (7.7)	22.5(7.1)	0.909**
Pain	0.862	9.5 (4.8)	9.4 (5.5)	0.886**
Urinarysymptoms	0.819	6 (3.2)	5.7 (2.9)	0.925**
Life quality impact	0.762	7.3 (2.3)	7.4 (2.5)	0.874**

**Correlation is significant at the 0.01 level. NIH-CPSI: National Institute of Health Chronic Prostatitis Symptom Index.

## 4. Discussion

Clinical studies using questionnaire forms are a preferred method in the objective evaluation of subjective complaints. The NIH-CPSI has been commonly used to evaluate symptom severity in patients with CP/CPPS. With the help of this well-prepared questionnaire, urologists have the chance to understand the past history of the disease and also to evaluate the response obtained as results of the treatment applied.

Like all other questionnaires, NIH-CPSI has also been translated into many languages, and its validation studies have been performed. To obtain an appropriate, convenient, and easily understandable form, each translation should be conducted by qualified, experienced translators. In our current study we worked with two independent translators during the four main steps of the process to prepare the Turkish version of NIH-CPSI from the original English form following the steps defined by Hutchinson et al. [15]. In the final stage, we conducted a pilot study with 15 patients.

Statistical evaluation of our findings demonstrated well that the Turkish version of the NIH-CPSI had excellent test-retest reliability (r [15] 0.909) and high internal consistency (Cronbach’s alpha = 0.864). Internal consistency was assessed in line with the original studies of Litwin et al. [1]. In the original questionnaire, nine items had high test-retest reliability (r: 0.83 to 0.93) and internal consistency (Cronbach’s alpha: 0.86 to 0.91).

Other validation studies regarding the application of NIH-CPSI, namely the Italian version (Cronbach’s alpha = 0.84–0.96), the Korean version (Cronbach’s alpha = 0.86–0.97), the Japanese version (Cronbach’s alpha = 0.83–0.87), the Brazilian version (Cronbach’s alpha = 0.85–0.93), and the Persian version (Cronbach’s alpha = 0.865) [11,12,16–18,] showed similar results with high level confirmation of the internal consistency.

Our study is not the first study regarding the use of NIH-CPSI or treatment of CP/CPPS in Turkey but our study is the first study on Turkish validation of NIH-CPSI. For example, although Arda et al. used the UPOINT classification according to NIH-CPSI scores in the treatment of CP/CPPS patients, no validation of NIH-CPSI in Turkish was mentioned in their study [19]. Again in the study performed by Sönmez et al. in 2011, the sexual dysfunction of CP/CPPS patients was included and the NIH-CPSI form was used. However, Turkish validation of NIH-CPSI was not used in this trial either [20].

Furthermore, despite numerous validation studies being conducted on NIH-CPSI none of them to date have focused on the correlation with VAS and IPSS questionnaires [11,13]. In light of this fact, apart from being the first Turkish NIH-CPSI validation study, our study has a distinct position from other studies considering the excellent correlation between the results of NIH-CPSI subgroups and also IPSS and VAS questionnaires (Pain-VAS correlation: 0.66, urinary symptom-IPSS correlation: 0.54). 

Our study had some potential limitations. First, the socio-cultural characteristics of the patients were variable and the number of participants in the control group was not as high as that in the CP/CPPS group. The socio-cultural differences, such as education and dialects of patients have also been shown to affect the understandability of the form [1,3,11]. Secondly, our study was not a multicenter one which might give further reliable data. 

## 5. Conclusion

In light of our current findings, we believe that the Turkish version of NIH-CPSI is a simple, feasible, and reliable form to be used with CP/CPPS patients. However, we believe that further clinical studies regarding the use of the Turkish version among Turkish patients need to be conducted in an attempt to increase the sensitivity of this test. Such studies will provide valuable relevant data to establish the profile of Turkish patients with CP/CPPS and also to determine the prevalence of cases with these symptoms in our country.

## Ethics committee approval

Kartal Dr. Lütfi Kırdar Training and Research Hospital Ethics Committee Unit 89513307/1009/300 (decision no. 8)

## Authors’ contribution

Concept and design: Assoc Prof Fatih TARHAN; resources: Alper COŞKUN MD, and Assoc Prof Fatih TARHAN; materials, data collection and processing: Alper COŞKUN MD, Övünç KAVUKOĞLU MD, and Prof K. Fehmi NARTER; analysis and interpretation: Alper COŞKUN MD, Utku CAN MD, Prof K. Fehmi NARTER, and Assoc Prof Fatih TARHAN; literature review: Alper COŞKUN MD, Utku CAN MD, and Övünç KAVUKOĞLU MD; translators: Assoc Prof Oktay AKÇA and Kubilay SABUNCU MD; writing the manuscript: Alper COŞKUN MD, Assoc Prof Fatih Tarhan, and Övünç KAVUKOĞLU MD; critical review: Utku CAN MD, Alper COŞKUN MD, and Prof K. Fehmi NARTER.
